# A scoping review of the perinatal healthcare experiences of Indigenous childbearing people

**DOI:** 10.1186/s12978-025-02220-w

**Published:** 2025-11-21

**Authors:** Rosina Darcha, Jill M. G. Bally, Shelley Spurr

**Affiliations:** College of Nursing, University of Saskatchewan, 107 Wiggins Rd, Saskatoon, Saskatchewan, S7N 5A4 Canada

**Keywords:** Indigenous peoples, Childbearing people, Perinatal healthcare, Healthcare experiences, Indigenous care workers and, Cultural safety

## Abstract

**Background:**

Globally, perinatal healthcare access, quality, and outcomes significantly vary between Indigenous and non-Indigenous childbearing people. This situation is precipitated by systemic barriers emanating from the longstanding effects of colonialization. Despite ongoing awareness of culturally safe perinatal care, Indigenous childbearing people continue to have challenging experiences. The purpose of this scoping review was to explore the perinatal healthcare experiences of Indigenous childbearing people to identify research gaps and inform future nursing/midwifery interventions to improve the challenges of engaging in perinatal healthcare in this population.

**Methods:**

The scoping review framework of Arksey and O’Malley was used in this study by searching, retrieving, and analyzing research papers from CINAHL, Ovid/Medline, PsycINFO, PubMed, and Web of Science.

**Results:**

Thirteen peer-reviewed articles published between 2002 and 2021 were analyzed. The experiences of Indigenous childbearing people who sought care during the perinatal period had their experiences classified into positive, negative, complex, and mediating. This scoping review reiterated the need for culturally safe healthcare, preferably delivered by Indigenous healthcare professionals in healthcare facilities situated in Indigenous communities.

**Conclusion:**

It is crucial to further explore the perinatal healthcare experiences of Indigenous childbearing people through in-depth qualitative research to develop culturally safe interventions, especially when life-limiting illnesses or life-threatening illnesses (LLIs/LTIs) occur.

**Implications:**

Overall, completion of this scoping review revealed the need for a comprehensive healthcare system transformation that addresses the needs of childbearing Indigenous families.

**Supplementary Information:**

The online version contains supplementary material available at 10.1186/s12978-025-02220-w.

## Background

Globally, perinatal healthcare access, quality, and outcomes significantly vary between Indigenous and non-Indigenous childbearing people [1]. Inequalities related to Indigenous perinatal healthcare access are complex [2, 3], and have been attributed to factors such as colonialism, the residential school system, discrimination, trauma, culture, and socioeconomic status [4–6]. Consequently, Indigenous peoples have been noted to have a deep-seated mistrust of healthcare systems, leading to decreased participation in healthcare [6]. Research has linked this suboptimal perinatal access to poor outcomes such as preterm births, stillbirths, and perinatal deaths [7, 8]. Westernized healthcare systems that are founded on colonial principles have inherent barriers that continue to impact individual perinatal and child health outcomes negatively [4]. These health disparities have precipitated Indigenous people in recent years, to advocate for the integration of culturally safe practices during the perinatal period, which spans from conception through to one-year post-delivery [9]. The concept of cultural safety in nursing was advanced by Irihapeti Merenia Ramsden in 2002 in New Zealand, based on Māori experiences [10]. Cultural safety from the healthcare recipient’s perspective is the decision of whether nursing services are safe for consumption, shifting the appraisal of healthcare from providers to recipients [10]. Irihapeti [10] argues that cultural safety is a matter of social justice, power, bias, and attitudes that affect nursing care, rather than the ethnicity or culture of patients or clients. The cultural safety framework seeks to address health system inequalities by examining concepts such as power, unconscious bias, and institutional racism [10]. Achieving cultural safety would require an understanding of the experiences of this population to develop responsive policies, interventions, and practices by healthcare professionals. A pragmatic step is to review the existing literature to identify gaps, challenges, and experiences of Indigenous childbearing individuals to guide such an understanding. This understanding would necessitate an in-depth evaluation of the existing literature to determine Indigenous childbearing people’s encounters accessing perinatal healthcare, the meaning of quality of care, experiences of discrimination, and perceived barriers to accessing perinatal care. Therefore, a scoping review of the perinatal healthcare experiences of Indigenous childbearing people was conducted.

## Methods

Rigorous approaches were adopted to explore the existing literature on Indigenous childbearing people’s experiences seeking perinatal healthcare. Hence, this study was guided by the framework endorsed by Arksey and O’Malley (2005) and includes the following stages: identification of the research question, identification of related studies, charting the data, and collecting, summarizing, and writing the findings [11]. The optional parallel consultation stage recommendation by Arksey and O’Malley [11] was not included because the main aim of conducting this scoping review was to identify knowledge gaps regarding the experiences of Indigenous childbearing people seeking perinatal healthcare in the existing literature, to inform nursing/midwifery practice, education, and policy development. These intentions align with the reasons for executing scoping reviews [11, 12].

### Stage 1: identify the research question

To resolve the persisting healthcare disparities confronting Indigenous peoples, several authors have concluded that there is an urgent need to transform mainstream healthcare services at all levels, including policies, physicians, nurses, midwives, support staff, and caring practices [4, 13]. Achieving this transformation would require credible data that describe Indigenous people’s actual perinatal healthcare experiences [9, 14]. Therefore, the purpose of this scoping study was to understand the perinatal healthcare experiences of Indigenous childbearing people. Using the population, concept, and context (PCC) framework recommended by the Joanna Briggs Institute (JBI) [12], the guiding question was as follows: What are the perinatal healthcare experiences of Indigenous childbearing people? This question was broadly framed to obtain depth of coverage of the experiences while retaining focus to guide the search strategy [11]. This carefully developed research question underpinned the systematic exploration of the perinatal healthcare experiences of Indigenous childbearing people to inform future research, education, and possible healthcare intervention development to improve practice. Table S1 outlines the PCC framework that guided this scoping review question (See supplementary file).

### Stage 2: identify relevant studies

Developing a thorough search strategy requires planning and expert collaboration to ensure the relevant literature is retrieved. Hence, the corresponding author collaborated with an experienced Health Sciences Librarian to identify relevant concepts and appropriate search terms [11, 12]. A comprehensive list of key search terms and Medical Subject Headings (MeSH) was developed. These were combined with the Boolean operators and used across multiple health science databases. This approach to search strategy development set the foundation for a systematic and replicable review process. Details of the search strategy are presented in Table S2 (See supplementary file). .

Using the developed search strategy presented in Table S2 (See supplementary files), five databases were searched, including Cumulated Index to Nursing and Allied Health Literature (CINAHL), Ovid/Medline, PsycINFO, PubMed, and Web of Science from July 19th to 21 st, 2024. The search was updated on August 6th and 7th, 2025, to ensure any new articles were captured, and a comprehensive search was completed. All these databases have relevant content to help answer the review question, ensuring a comprehensive depth and breadth of the subject area is achieved [11, 12, 15]. To ensure that all relevant studies are captured in this review, an extensive search from the inception of the five databases was done. A search of reference lists, hand searching of related journals, conference resources, Indigenous-specific databases such as the Indigenous Studies Portal (iPortal) of the local university, and website searches were conducted. However, articles found in these searches were already retrieved from the extensive search of the databases [11]. Again, the search for appropriate articles was also guided by clear inclusion and exclusion criteria [11, 12, 15]. Articles included were peer-reviewed and published in the English language; either quantitative, qualitative, or mixed-method in nature; focused on the experiences of Indigenous childbearing peoples during pregnancy, birthing, or postpartum; and the setting of the experiences was either in hospitals, clinics, or community health settings. Articles were excluded if they were not written in English or had an English translation; existing literature reviews, studies focused on non-Indigenous childbearing people, birthing outcomes, and studies in a non-healthcare setting (See Table S3 in the supplementary file).

### Stage 3: study selection

A systematic search and screening process to identify relevant studies on the perinatal healthcare experiences of Indigenous childbearing people, using five databases and a joined screening approach was applied. The inclusion and exclusion criteria outlined in Table S2 were strictly observed. The search yielded 10,189 articles across all the databases. These articles were exported from EndNote to the Rayyan software, as recommended by Valizadeh, Moassefi [16], to facilitate a systematic screening process. The other authors (JB and SS) were invited to Rayyan as collaborators.

**Fig. 1:**
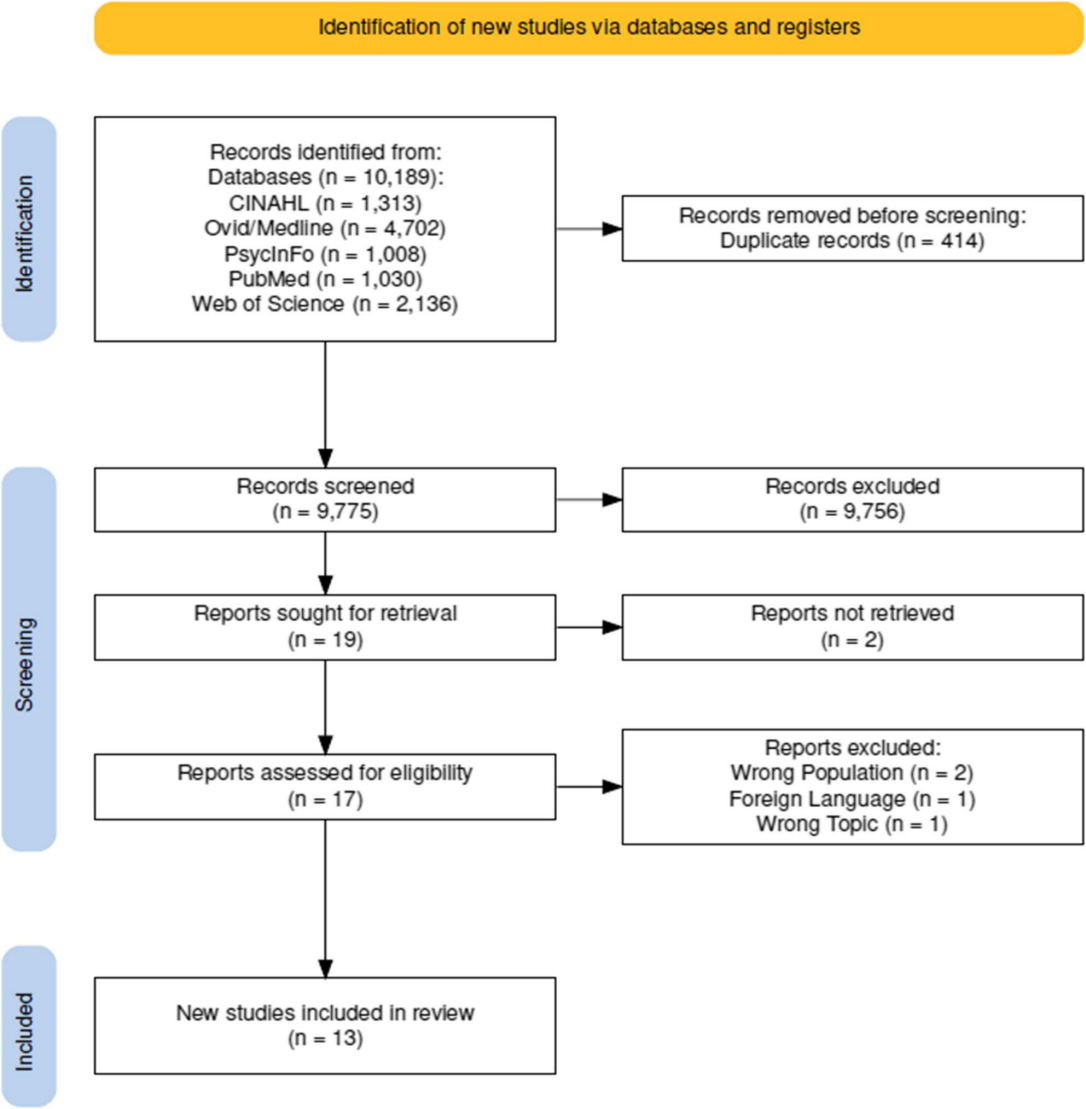
PRISMA Flow Chart of the Study Section Process. Source: Developed using the Shiny app for making PRISMA2020 flow diagrams [17]

An initial data-cleaning process identified 775 possible duplicates. These potential duplicates were examined, and 414 articles were confirmed as duplicates and deleted, leaving 9,775 articles for the initial screening. The first author screened the titles and abstracts of the articles while consulting the other authors when needed, ensuring that two of the authors reviewed all articles. This process resulted in the exclusion of 9,756 articles. Thirty-six articles were either included or marked for additional review in the ‘maybe’ folder in Rayyan when the alignment of the article was uncertain for inclusion. To ensure an unbiased study selection process, all three authors screened the 36 articles with Rayyan’s blind feature enabled. The blind was lifted when this phase was completed. The authors met to discuss the articles selected based on the inclusion and exclusion criteria, resulting in 19 articles being selected for full-text screening. Following Peters, Godfrey [12] guidelines, the authors met to discuss the inclusion or exclusion and the rationale for the decisions after completing the full-text screening. Based on consensus, 13 articles were selected and analyzed for this scoping study. Figure 1 is a PRISMA flow chart that illustrates all the stages and the number of articles included or excluded.

### Stage 4: charting the data

The extraction and charting of data followed an organized process, utilizing a modified Pinch table to systematically document and analyze the relevant extracted data. In the fourth stage of the Arksey and O’Malley [11] framework for scoping reviews, data were extracted using a structured charting form. The team adapted the Pinch table, a comprehensive data organizational tool introduced by Pinch [18]. This tool presents a rigorous framework for capturing, organizing, and analyzing the data from the 13 articles. The modified Pinch table was used to organize the 13 articles under the following headings: author/year/country; purpose/aim, population/sample; methodology/methods; findings; implications; and future research. To ensure comprehensiveness, the first author completed the initial data extraction. This was followed by several reviews and validation by the other two authors, forming an overview of key data from all the selected studies (See Tables S4a and S4b in the supplementary file). This systematic data charting process ensured the extraction and analysis of relevant data from the included studies, based on which patterns, themes, and gaps in the literature were identified. This process of making meaning of the data extracted did not apply the narrative tradition suggested by [11]. We adopted the analytical process used in recent scoping reviews by [19–21]. This process involves categorization and description of the data to answer the scoping review question [19–21].

### Stage 5: Collating, Summarizing, and reporting the results

**Fig. 2:**
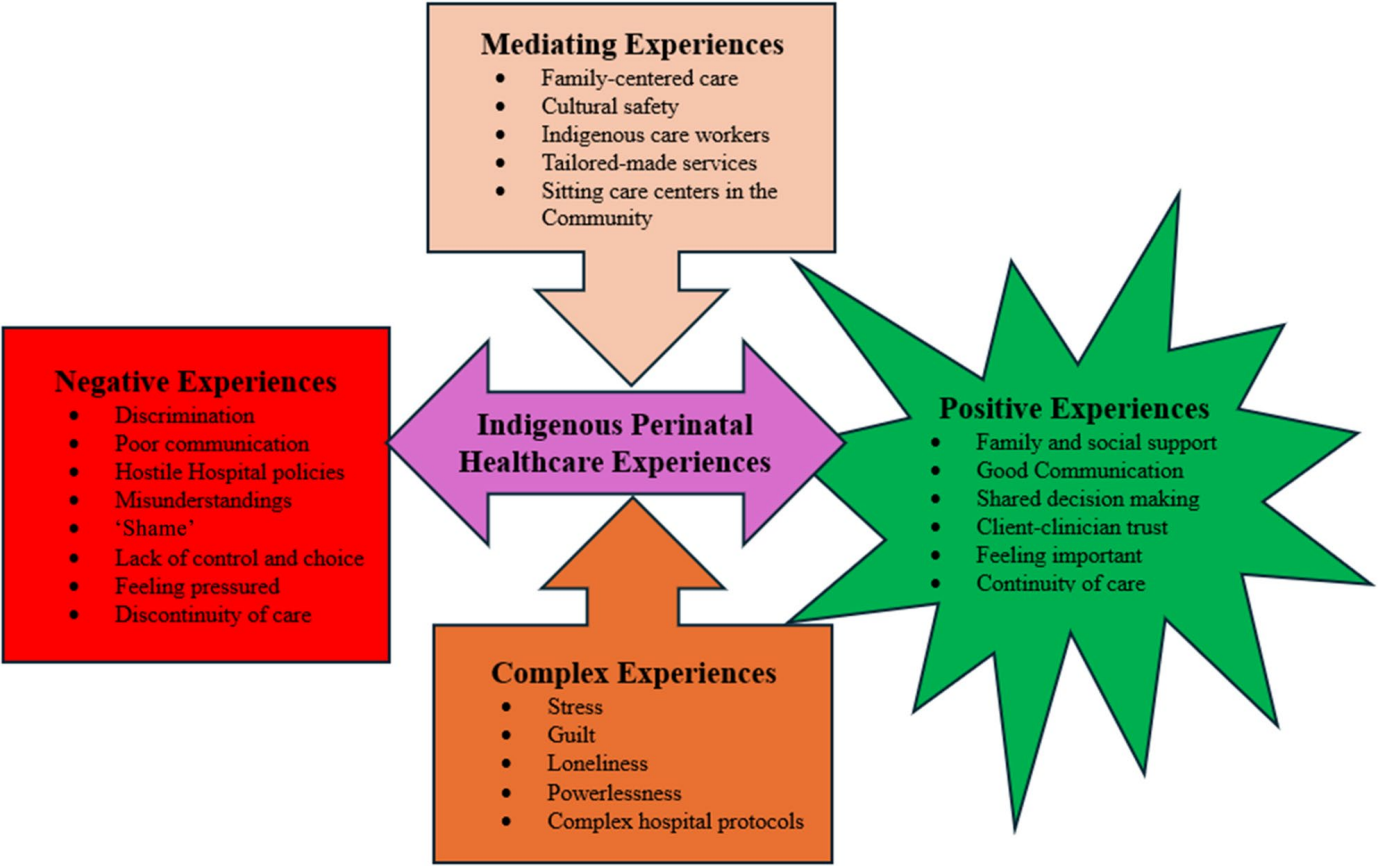
A Conceptual Model Summarizing the Scoping Review Findings. Source: Authors’ Construct (2024)

In this stage, the authors organized and presented the findings without assessing the quality of the articles included, given that this is not necessary for scoping reviews [11, 12, 19]. All authors collaboratively conducted the data analysis by grouping and describing relevant concepts that emerged from the extracted data. This was achieved by following the inductive approach outlined in [21]. The steps adopted include reading and re-reading the extracted data, open coding, developing a coding framework, organizing the extracted data under the coding framework, and categorizing and developing an overarching framework [21]. The categorization of the concepts revealed the existing relationships between them (See Fig. 2), depicting the key patterns in the Indigenous birth givers’ perinatal healthcare experiences outlined by the studies included in this scoping review. This collaborative process of collating, summarizing, and reporting the findings from the 13 studies resulted in a narrative that answered our research question and identified implications for nursing and midwifery research, policy, and practice [15]. Finally, all three authors collaborated in drafting and reviewing the final report.

### Rigor

Several steps were taken to ensure rigor in conducting this scoping study. These include involving a Health Sciences Librarian from the outset to ensure a rigorous, valid, and replicable search strategy. Performing a multi-phased screening process in Rayyan with the other two co-authors (JB and SS) as collaborators ensured a non-biased process. The authors held meetings after completing each step of the screening process to discuss the independent selections made. This resulted in an unbiased selection of the 13 articles that explored the perinatal healthcare experiences of Indigenous childbearing people. Furthermore, rigor was maintained by observing the guidelines in the Preferred Reporting Items for Systematic Reviews and Meta-Analyses (PRISMA) Extension for Scoping Reviews (PRISMA-ScR) [22]. Adhering to appropriate methodological frameworks and guidelines safeguards a systematic method of reviewing existing literature while maintaining academic rigor.

## Findings

The scoping review completed permitted the exploration of 13 studies published between 2002 and 2021 related to perinatal healthcare experiences of distinct Indigenous childbearing people across four countries [3, 23–34]. The term “Aboriginal” was used to represent Indigenous peoples from Australia. In Canada, Indigenous peoples include the First Nations (North American Indian), Métis, and Inuit peoples. For this article, the general term “Indigenous” was used apart from the findings from Australia. Most of the studies were conducted in Australia, 61.5% (*n* = 8). The rest were conducted in Canada (*n* = 3), New Zealand (*n* = 1), and the United States of America (*n* = 1). The studies explored the experiences of the following Indigenous populations: Australian Aboriginal and Torres Strait Islander people [25–30, 32, 33], the Māori people of New Zealand [23], the Mi’kmaq, Haida, Kwakwaka’wakw, and Nuxalk First Nations people of Canada [3, 31, 34], and the American Lumbee Indians [24]. The population mainly studied was women/childbearing people 76.9% (*n* = 10) [3, 25–29, 31–34]. Additional studies included 15.4% (*n* = 2) women/parent-infant dyads [24, 30] and 7.7% (*n* = 1) family unit [23].

Methodologically, the majority, 84.6% (*n* = 11), used qualitative designs, employing semi-structured interviews, and various Indigenous research approaches. The remaining 15.4% (*n* = 2) studies used population-based quantitative methods. Of these two quantitative studies, Brown, Gartland [30] adopted the Measure of Indigenous Racism Experience (MIRE), a tool for assessing self-reported racism developed by Paradies and Cunningham [35]. However, the other study by Brown, Weetra [29] did not explicitly specify the tool used for data collection. The studies highlighted a range of perinatal healthcare experiences, including positive, negative, and mediating, with a few studies presenting additional complexity of life-limiting illnesses (LLIs) or life-threatening illnesses (LTIs) such as preterm births [23, 24].

### Positive experiences

Indigenous childbearing people recounted different levels of satisfaction with perinatal care, with positive experiences strongly linked to cultural sensitivity, being made to feel special, and holistic support systems [27, 28, 32]. Seear, Spry [27], Watson, Hodson [28], Homer, Foureur [32] documented particularly encouraging experiences, while Brown, Fereday [25] found mainly physical and clinical care satisfaction, with emotional needs often unmet. Support emerged as vital to the positive experiences of Indigenous childbearing people. These supports were from either the family, community, or healthcare workers [23, 25–28, 32–34]. Indigenous healthcare workers such as midwives were noted to improve these experiences [26, 29, 32], as did good client-clinician communication, being made to feel important, and shared decision-making were highly recommended [25–28, 32, 33]. These participants described good communication and shared decision-making as being included in clinical discussions using simple, clear language, face-to-face communication, and receiving regular updates on what is happening in their care to make informed decisions. The Aboriginal Family Birthing Program (AFBP) and Malabar Community Midwifery Link Service, both Australian initiatives that ground perinatal care in Aboriginal culture, were cited as examples of tailor-made perinatal services. This is because participants said they received culturally safe care, had a trusting relationship with care providers who advocated for them, continuity of care, and easily accessible care that successfully integrated traditional practices and provided much-needed social support [29, 32].

These positive findings demonstrate that perinatal healthcare offered through culturally grounded ways, relationship-centered care, and prioritizing Indigenous voices, values, and traditions not only enhance care satisfaction but also serves as an ideal model for transforming healthcare delivery that facilitates equitable and respectful perinatal healthcare for Indigenous people.

### Negative experiences

Indigenous participants faced substantial perinatal healthcare challenges in the form of systemic discrimination, cultural insensitivity, and communication barriers [30, 31, 34]. According to the studies, the Aboriginal childbearing people encountered racism and discrimination [30, 31, 34, 36], which was attributed to the medical staff’s poor cultural awareness [25, 28]. This limited cultural insight was demonstrated by hostile hospital policies, which create an environment that marginalizes Indigenous childbearing people’s traditional practices [23, 26, 31]. Communication breakdowns were common, and healthcare workers’ lack of cultural awareness fostered misunderstandings that caused fear among Indigenous clients [3, 25, 28, 33], adversely affecting their access to perinatal care [3, 25, 29, 30]. Furthermore, the experience of ‘shame’ emerged as a deterrent to Indigenous childbearing people’s engagement with healthcare systems [26, 28, 33]. These systemic challenges directly impacted health outcomes, as discrimination was linked to low birth weight among the participants [30]. Other compounding factors were structural inequalities like staff shortages, limited Aboriginal healthcare providers, and broader socioeconomic health disparities [27, 29–31, 34]. Brown, Varcoe [31], Minniecon, Parker [33], and Varcoe, Brown [34] reported that Indigenous childbearing people described perinatal services that were discontinuous and characterized by a lack of control and choice. The participants said health workers were trying to be helpful, but were experienced as very ‘pushy’ [33]. Geographical barriers to care, led to forced evacuation, often separating birth givers from their families, loved ones, and other children for long durations while seeking perinatal healthcare [31, 34].

The barriers experienced by the childbearing people in the studies included in this scoping review, ranged from discriminatory attitudes and a lack of cultural safety to geographical isolation and systemic inequalities, collectively creating a healthcare atmosphere that potentially compromised their physical and mental health outcomes.

### Complex experiences

Indigenous childbearing people going through high-risk perinatal complications, such as preterm births, experience emotional and health system challenges that disrupt traditional caregiving. Researchers including Burns, Whitty-Rogers [3], Brown, Weetra [29], noted that increased stress levels during such complications are associated with perceived discrimination. Perceived discrimination adversely affected healthcare experiences during these unexpected upheavals [23, 24]. To cope with the chaos at this time, Indigenous parents tend to rely on cultural practices such as burying the placenta, ‘smudging’, and traditional medicines [23, 27], which were often not supported by health facility policies [23, 24, 27].

Indigenous parents in the Neonatal Intensive Care Unit (NICU) reported several challenges, including: Navigating complex hospital admission protocols, receiving inadequate medical information, and managing contradicting clinical directives [23, 24]. Also, parents’ ability to bond with their infants was hindered by emotional disturbances like loneliness, fear, frustration, powerlessness, and guilt [23, 24]. This is worsened by restrictive hospital policies that prevent holding their babies in the NICU and restricted visiting hours [23, 24, 31]. According to Brooks, Holdtich-Davis [24], some parents developed symptoms of post-traumatic stress disorder (PTSD) that persisted after discharge from the NICU. Brown, Varcoe [31] and Varcoe, Brown [34] described participant experiences of feeling stressed, depressed, and in some cases, developed postpartum depression. Caregivers said they often failed to perform important cultural newborn ceremonies like naming and navigating a balance between traditional and Western medical practices [24].

The combination of high-risk perinatal complications and systemic healthcare barriers led to a dual crisis for Indigenous families who participated in the research included in this scoping review [23, 24]. Medical emergencies not only threatened physical health but also disrupted important cultural practices and traditional healing ways, leaving parents to navigate trauma and recovery within an unsupported environment.

### Mediating experiences

Through the completion of this scoping review, it was revealed that globally, there is an urgent need for healthcare systems that are culturally safe and responsive to the perinatal healthcare needs of Indigenous childbearing people. To achieve this, participants made suggestions that could enhance positive experiences or resolve negative or complex experiences during the perinatal period. Participants across all the studies wanted healthcare providers to acknowledge and respect their cultural identity [24], thereby providing perinatal care that is culturally safe and appropriate for their needs [3, 23–30].

Such a model of Indigenous-appropriate perinatal care to promote access could only be developed by seeking the views of Indigenous childbearing people, Elders, and community members [3, 28, 31]. Training clinicians to comprehend cultural safety [3, 27, 30] would help them understand the health experiences of Indigenous childbearing people [26]. Fostering health professionals’ cultural understanding would enable trusting relationships with Indigenous clients, which emerged in the literature as key to positive experiences of care [23, 26, 27, 32]. Furthermore, it was documented that training and recruiting Indigenous midwives or perinatal care workers to care for Indigenous childbearing people was noted as vital to culturally safe care [25, 27, 29, 32]. Siting perinatal health facilities in Indigenous communities has been presented as a feasible solution to improving perinatal care experiences [28]. Watson, Hodson [28] also suggested closing the gap due to the social determinants of health inequalities by offering resources tailored to the needs of the targeted Indigenous population.

These improvements can only be achieved by filling the knowledge gap through future research evidence that seeks to understand the nuances of Indigenous cultures, spirituality, lifestyle, healthcare experiences, and discrimination. This is critical to minimizing some of the challenges noted in this scoping review. Generating such knowledge would, ultimately, inform policies that promote the integration of Indigenous cultures into perinatal care [3, 23–27, 30]. Perinatal care models that meet the needs of Indigenous childbearing people could be developed by promoting cultural understanding, Indigenous-led research, and strategies to address systemic barriers to equitable perinatal care. To potentially transform perinatal healthcare for Indigenous families, models of care that are Indigenous-led and community-centered are required. According to the literature reviewed, such a model of care must include approaches that honor traditional knowledge systems, address structural inequalities, be grounded in cultural safety, and yet al.so respect the self-determination priority of Indigenous communities [3, 23–30].

The salient concepts and their relationships, described in the findings above, have been summarized by the authors into a conceptual model, illustrated in Fig. 2. This conceptual model is the outcome of the authors’ analysis and subsequent understanding of the data extracted from the studies included in this scoping review.

## Discussion

The purpose of this scoping review was to identify the knowledge gap in the literature regarding the perinatal healthcare experiences of Indigenous childbearing people. The authors conducted a broad search and included 13 studies that met the inclusion and exclusion criteria. The included studies provided valuable insight into understanding this important topic. Findings from the literature revealed that participants had interrelated positive, negative, complex, and mediating encounters with the healthcare system and professionals. This evidence highlights the need for future nursing/midwifery research, interventions to improve nursing care practices, and education or training on cultural safety. These results illustrate the multifaceted nature of the perinatal healthcare encounters of Indigenous birth givers and health professionals [37, 38].

The positive experiences of participants, although relatively less common occurrences, present strategies for health workers to enhance perinatal care for Indigenous childbearing people. The ability to recognize and respect Indigenous cultural identity was the foundation of the participant’s positive experiences. Key to positive experiences is promoting support from family and loved ones; building trusting relationships with clinicians; providing culturally safe care; good staff-client communication that ensures clients’ full participation in their care; and training and recruiting Indigenous health professionals conversant with Indigenous childbearing culture. Although these approaches embody care that is culturally safe for Indigenous childbearing people, none of the studies specifically outlined how to achieve these strategies. For example, Brown, Weetra [29] quantitative study proved that the AFBP was culturally safe for Indigenous childbearing people without the depth that a qualitative study would have provided. Similar results were found in earlier studies that noted increased client care satisfaction and access when perinatal care was made culturally safe by incorporating Indigenous practices and care workers in the delivery of services [39, 40]. Nevertheless, this scoping review has illuminated the need to delve deeper to understand how to build trusting relationships or provide culturally safe perinatal care, rather than enumerate what should be done, as is often the case in related studies.

Negative experiences were more prevalent in many of the studies reviewed, and these were described as discrimination, marginalization, racism, lack of Indigenous cultural and spiritual insight, communication barriers, and hostile hospital policies. These challenges are remnants of racism and colonialism persisting in healthcare systems [41]. Other studies have confirmed that Indigenous people endure adverse perinatal experiences like unfair treatment and discrimination [2, 42]. According to Chowdhury [43], the Indigenous childbearing people of Garo reacted to racist experiences by avoiding hospitals as a way of expressing their resistance to racism. In Canada, for example, the persistent access barriers, exacerbated by negative experiences, despite the Truth and Reconciliation Commission’s (TRC) calls to action numbers 18 to 24 on health [44], suggest the existence of considerable implementation gaps in providing culturally safe care for Indigenous people. The TRC of Canada made 94 calls to action to repair the atrocities that occurred for over 100 years in Indian Residential Schools. These calls to action target various sectors of society, of which 18 to 24 target improving the health of Indigenous people in Canada [44]. Following these calls to action, the Government of Canada has implemented several national and provincial programs to improve the perinatal healthcare of Indigenous people [45]. Despite the substantive investment to this effect, corresponding outcomes have not been achieved [46]. The minimal positive results obtained may be explained by the findings from this scoping review, which suggest that the programs were probably implemented without adequate Indigenous consultation and research evidence to guide the processes. This is confirmed by finding a limited number of primary studies that explored the perinatal healthcare experiences of Indigenous people in Canada [22]. Therefore, this scoping review not only confirms the existence of profound negative perinatal healthcare experiences but also highlights a unique population that is yet to benefit from extensive research evidence on perinatal healthcare.

The complex perinatal experiences are obscure in the literature, yet are vital determinants of Indigenous childbearing people’s experiences. In this scoping review, complex experiences identified underscore the additional stressors when LLIs/LTIs such as preterm births occur, defying simple classification of the experiences as positive or negative. This compounds healthcare experiences, ultimately suggesting that some positive experiences could degenerate into negative ones and negative ones may worsen over time. The available related literature only explored the prevalence, risk factors, and outcomes of LLIs/LTIs among Indigenous people [1, 47, 48], with few studies examining participants’ experiences during the perinatal period [23, 24]. It is, therefore, important to explore these experiences in-depth. Specifically, exploring when LLIs/LTIs occur is even more critical because the client-clinician interaction is prolonged, hence the need for robust and practical culturally safe interventions. Health workers can only provide this needed support if adequate data exists to guide the delivery of perinatal healthcare for this unique population.

Mediating experiences emerged as transformational, an intersection between positive and negative experiences. Mediating experiences in this literature review are bidirectional and capable of changing the direction of perinatal healthcare experiences from negative to positive and vice versa, as envisioned in Fig. 2. It is encouraging that mediating factors emerged from the literature to guide stakeholders’ ability to transform the perinatal healthcare landscape of Indigenous childbearing people. The crux of the matter is recognizing Indigeneity, developing inclusive care models, and delivering perinatal care in a culturally safe manner. Two studies have similar findings [2, 39] and recognize the role of intermediaries such as Indigenous midwives, doulas, and cultural support workers of Indigenous origin working in either mainstream health facilities or in the community as transformative approaches to converting negative experiences into positive ones [39]. Despite these similar results, the findings of this scoping review are unique because they present a potentially clear and holistic picture of the perinatal healthcare experiences of Indigenous childbearing people. Not only was the need to further explore the perinatal healthcare experiences of Indigenous childbearing people highlighted, but also the critical nature of understanding the experiences of this population when LLIs/LTIs occur.

## Strengths and limitations

Several limitations should be considered when interpreting the findings of this scoping review. First, the studies included were qualitative and quantitative, excluding theoretical literature. Thus, important theoretical perspectives could have been excluded. Second, the inclusion of only articles published in the English language could have excluded valuable literature published in other languages. Third, it is noteworthy that eight of the 13 studies included were conducted in Australia, indicating that the experiences described might differ culturally from those in the other three countries, limiting the transferability of some findings. However, it is likely that readers will find meaning and usefulness from review of the findings of this scoping review. The results from the 13 studies included in this review might not be enough to comprehensively depict all the perinatal healthcare experiences of Indigenous childbearing people seeking healthcare. Finally, it is also possible that relevant articles may not have been included even though a thorough search was completed with the assistance of an experienced health sciences librarian. However, the findings are still valuable and provide a basis for future research to inform nursing and midwifery education, research, policy, and practice.

## Conclusion

This scoping review explored the perinatal healthcare experiences of Indigenous childbearing people globally. Thirteen articles that overtly described the participants’ healthcare experiences were analyzed. The gap in the limited number of published articles on this important research topic, despite a thorough literature search, reiterates the need for this scoping review. Individually, each study outlined the perinatal healthcare experiences from multiple diverging perspectives. However, this scoping review organized the results into four categories: Positive, negative, complex, and mediating experiences. Organizing the results from the 13 included studies into these categories led to findings that are unique, holistic, and provide an understanding of the experiences of Indigenous childbearing peoples. Furthermore, the findings offer potential solutions to improve these experiences by emphasizing cultural safety. This implies recognizing and integrating cultural safety practices into mainstream perinatal healthcare; hence, the need for healthcare systemic changes. The preference for culturally safe care may necessitate an increased representation in the Indigenous healthcare workforce. Formulating meaningful policies, interventions, and training a capable Indigenous workforce would require knowledge and understanding of these experiences through an in-depth qualitative study to generate data that could be directly applied to practice. Finally, a hidden but critical area that emerged from the literature is the complex experiences associated with the presence of LLIs/LTIs during the perinatal period. These experiences defy classification yet are powerful enough to consider changing the direction of the perinatal healthcare experiences of Indigenous childbearing people. Overall, these findings are all compelling in answering the scoping review question and highlighting the crucial need for an in-depth qualitative exploration of this study area.

## Supplementary Information

Supplementary Material 1.

## Data Availability

No datasets were generated or analysed during the current study.

## Abbreviations

AFBP: Aboriginal Family Birthing Program
CINAHL: Cumulated Index to Nursing and Allied Health Literature
JBI: Joana Briggs Institute
LLIs: Life-Limiting Illnesses
LTIs: Life-Threatening Illnesses
MeSH: Medical Subject Headings
NICU: Neonatal Intensive Care Unit
PCC: Population Concept and Context
PRISMA-ScR: Systematic Reviews and Meta-Analyses (PRISMA) Extension for Scoping Reviews
PTSD: Post-traumatic stress disorder
TRC: Truth and Reconciliation Commission
