# Supplementary material for: A scoping review of the perinatal healthcare experiences of Indigenous childbearing people
Source: Reprod Health. 2025 Nov 21;22:264. doi: 10.1186/s12978-025-02220-w (PMC12750894; doi:10.1186/s12978-025-02220-w)
Supplement: Supplementary file 1 — Supplementary Material 1. [file 12978_2025_2220_MOESM1_ESM.docx]

**Table 1**

*PCC Framework*

| **PCC Framework** | **Question** | **Keywords/Key Terms** |
| --- | --- | --- |
| Population | Indigenous childbearing people who are pregnant, are in labor, or have given birth (up to one year after birth) | Indigenous  Indigenous people  Aboriginal  First Nations  Native  Inuit  Métis  American Indian  Family  Ancestry  Lineage  Parent  Birth  Relation  Mother  Women  Pregnant women  Pregnant people  Childbearing people  Expectant mothers  Pregnant individuals  Gestational parents  Expectant parents  Birthgivers |
| Concept | Experiences related to childbearing | Healthcare experiences  Maternal health  Midwifery care  Pregnancy care  Antenatal care  Prenatal care  Perinatal care  Postnatal care  Obstetric care  Labour  Reproductive health care  Maternity care  Birthing support  Gestational health  Pregnancy support services  Childbirth education  Family planning services  Fertility services  Parental health care  Patient Satisfaction  Client satisfaction  Access to care  Quality of care  Cultural safety |
| Context | Healthcare settings that provide care to Indigenous childbearing people | Hospitals  Clinics  Community health centers  Traditional healing practices |

**Table 2***A list of Key Search Terms, MeSH Headings, and Boolean Operators Used*

| **Search Strategy:**   1. (Indigenous or Aboriginal or "First Nations" or Native or Inuit or Metis or "American Indian").mp. 2. exp Indigenous Peoples 3. Famil* or ancestr* or lineage or parent* or birth* or relat* 4. 1 or 2 or 3 5. (Mother* or Women or Pregnan* or "Expectant mother*" or "Childbearing people" or "Gestational parent" or "Gestational parents" or "Expectant parent*" or Birthgiver* or matern* or antenatal or postnatal or birthing).mp. 6. Pregnant Women 7. pregnancy/ or labor, obstetric/ or parturition/ or placentation/ or pregnancy in adolescence/ or exp pregnancy outcome/ or pregnancy, high-risk/ or pregnancy maintenance/ or exp pregnancy, multiple/ or pregnancy, unplanned/ or pregnancy, unwanted/ 8. 5 or 6 or 7 9. (experience or "Patient satisfaction" or “Client satisfaction” or "Access to care" or "Quality of care" or "Cultural safety" or "Cultural care" or "Cultural competence").mp. 10. exp "patient acceptance of health care"/ or patient satisfaction/ or patient preference/ or exp treatment refusal 11. 9 or 10 12. ("Maternal health" or “Midwif* care” or "Pregnancy care" or "Antenatal care" or "Prenatal care" or "Postnatal care" or "Obstetric care" or "Reproductive health" or "Maternity care" or "Birthing support" or "Gestational health" or "Pregnancy support service" or "Childbirth education" or "Family planning service" or Fertility service* or "Parental health").mp. 13. Maternal Health 14. 12 or 13 15. 4 and 8 and 11 and 14 |
| --- |

**Table 3**  *Inclusion and Exclusion Criteria of the Scoping Review*

| **Criterion** | **Inclusion** | **Exclusion** |
| --- | --- | --- |
| Language | Studies published in the English language or have available English translations. | Languages other than English or without English translations |
| Period | From the inception of the databases | No time limits |
| Type of Articles | Peer-reviewed primary studies, Qualitative, Quantitative, or Mixed methods studies, Government Reports, Policy Documents, and Indigenous-specific databases. | Existing literature reviews |
| Study Focus | Studies focused on perinatal healthcare experiences (professionals or services) of Indigenous childbearing people from any country, experiences during pregnancy, giving birth, or during the puerperium or postpartum (period up to one year after birth) | Focused on non-Indigenous child-bearing people; child-bearing health outcomes without describing healthcare experiences |
| Study Setting | Settings of the experiences or interactions where childbearing services are provided, such as hospitals, clinics, and community health centers | Setting in a non-healthcare context |

**Table 4a***Summary of Qualitative Studies Included in the Scoping Review*

| **Author and**  **Country** | **Purpose/Aim** | **Population/**  **Sample** | | **Methodology/**  **Methods** | | **Findings/Healthcare Experiences** | | **Implications** | **Areas for Future Research** |
| --- | --- | --- | --- | --- | --- | --- | --- | --- | --- |
| Adcock et al., (2021)  New Zealand | To voice the experiences, views, and attributions of Whānau (family collectives) of preterm Māori infants. | 19 Whānau (19 mothers, 9 fathers, and 13 non-parents Whānau) | | Interpretative Phenomenology and Kaupapa Māori (by, with, for Māori) using focused life story interviews | | Four themes emerged, describing the time spent at the Neonatal Intensive Care Unit (NICU) with their preterm babies as chaotic and lonely due to separation from their babies and family. | | There is an urgent need for culturally safe care by listening to caregivers; involving families in care decision-making; recognizing caregivers’ expertise; and fostering good clinician-client communication. | An in-depth exploration of preterm birth experiences of families and exploring Māori-centered neonatal care models. |
| Brooks et al., (2016)  United States of America | To explore parenting premature infants within the context of culture, birth, and hospital experiences | 17 American Indian mothers and their premature infants | | Longitudinal, descriptive qualitative design using semi-structured interviews | | Three themes highlight diverse interactions between birth givers and clinicians in the NICU. Participants felt unprepared among other numerous emotional stressors affecting the maternal role development. Helpful factors include cultural identity, spirituality, and cultural practices. | | Insights from this study indicate that childbearing people experience adverse effects such as parental role disturbance and symptoms of long-term posttraumatic stress due to the admission of their preterm infants to the NICU. Mitigating factors during this time are cultural practices and communication. | Exploring the integration of cultural factors in the care of premature infant care and the communication practices of healthcare professionals. |
| Brown et al., (2016)  Australia | To explore the experiences of Indigenous women's access to care during labor and birth at a tertiary public hospital. | 14 Indigenous women | | Qualitative interpretive Heideggerian phenomenology using semi-structured interviews | | The authors found six themes of varied experiences, highlighting the resilience and strength of birth givers in their quest to ensure the health of their infants. Participants indicated that physical care from clinicians was positive but lacked emotional support. Good or poor communication was identified as the main determinant of their care experiences. | | Participants experienced a lack of culturally safe care due to discrimination in mainstream hospitals. Consequently, culturally safe midwifery care and good communication that involves clients and families in care decision-making positively impacted perinatal care experiences. | Exploring cultural planning and integration in maternity care. |
| Burns et al., (2019) Canada | To understand Mi'kmaq women's experiences accessing prenatal care in Nova Scotia. | 4 Mi’kmaq women | | Qualitative description and the Two-Eyed Seeing approach using semi-structured interviews | | The study identified three themes that described barriers to accessing prenatal care by rural Indigenous birth givers such as long traveling distances and transportation challenges. They associated positive experiences with strong social support, cultural safety, and holistic care (body, mind, and spirit). | | Indigenous childbearing people require interventions to overcome challenges with prenatal care access due to socioeconomic factors. Culturally appropriate care is necessary to improve care utilization and better perinatal outcomes. | Exploring Mi'kmaq Elders and community perspectives of perinatal services to inform policy and improve access to care. |
| Kelly et al., (2014)  Australia | To explore Aboriginal childbearing people’s Continuity of Care (COC) experiences with Aboriginal midwifery students. | 4 Aboriginal and Torres Strait Islander women. | | Qualitative Exploratory, Descriptive design guided by Indigenous research approach using semi-structured interviews. | | The participant’s experiences were grouped into four themes. Positive experiences were related to communicating without feeling ‘shame', good student-client relationships, and receiving the needed support. However, healthcare systemic challenges negatively impacted participants’ experiences. | | The services of Aboriginal midwifery students enhanced continuity of care which could improve health outcomes for Aboriginal families and help to overcome barriers to accessing healthcare services. It is vital to increase the number of Aboriginal midwives. | Exploring strategies to align connectedness and professional boundaries in midwifery services. |
| Seear et al., (2021)  Australia | To describe Aboriginal women's experiences of antenatal care in the Kimberley. | 124 Aboriginal women | | Qualitative Descriptive Approach utilizing standalone interviews | | Participants mostly had positive antenatal care experiences divided into five key themes that stressed the importance of building trust in clinician-client relationships, health assessment, family support, simplifying health information to the client's level, and mitigating the challenges of traveling or relocating to access care. | | The study generated evidence to improve the quality of care based on the experiences of women. The study concluded that training Aboriginal healthcare staff to deliver culturally safe care is crucial, and expanding support for women who travel to access care is needed. | Exploring cultural practices like smoking ceremonies and bush medicines. |
| Watson et al., (2002)  Australia. | To develop educational  resources based on participants' and healthcare professionals' experiences. | 12 Aboriginal and Torres Strait Islander women | | Descriptive  Qualitative Study employing informal 'chat' style interviews | | The experiences were mixed, including a lack of preparation for preterm birth, unknown hospital admission expectations, loneliness due to relocation, accommodation challenges, misunderstandings with staff, and missing important cultural ceremonies, among others were participants’ experiences. | | Findings revealed challenges with clients understanding medical terminologies and Western bureaucracies They expressed their desire to access birthing-related care in their communities. Also, it emerged that clinicians lacked a basic understanding of Indigenous culture. | None suggested. |
| Brown et al, (2011)  Canada | This study explored the birthing experiences and traditional  birthing practices among the Haida, Kwakwaka’wakw, and Nuxalk  First Nations | 125 (102 mothers, 3 fathers, 5 youths, 11 elders, and 4 healthcare workers) from the Haida, Kwakwaka’wakw, and Nuxalk  First Nations. | A community-based participatory and ethnographic  design using individual interviews, focus groups, and participant observations | | The rural-dwelling Indigenous childbearing people indicated that their perinatal healthcare experiences were characterised by economic and geographical challenges, cultural disruption, impact of colonization, lack of control and choice, care discontinuity, racism and discrimination, forced evacuation, hospital restrictions, acts of resistance and self-advocacy, and stress and depression. | | Key nursing implications are:  Nurses should reflect on how their social circumstances affect their response to Indigenous people; understand how medical colonialism is a social determinant of health; go beyond cultural appropriateness to provide culturally safe care; facilitate Indigenous peoples' choice and control; challenge disempowering dominant practices; integrate traditional knowledge into biomedical care; develop rural lens; and engage in reflective practice. | | None suggested |
| Homer et al., (2012)  Australia | To report the evaluation from the perspective of the women who  accessed the services of Malabar | 353 women who gave birth through the Malabar service during 2007 and 2008. An Aboriginal Women’s Evaluation Group of local women, community elders,  and mothers. | A descriptive study using qualitative approaches | | The overall experience described by Aboriginal and Torres Strait Islander women who accessed care from the Malabar midwifery link services was: feeling valued, supported, and receiving culturally appropriate care that respected their needs while providing access to the necessary medical resources when required. | | The study highlighted the need for more community-based appropriate maternity services. Also, further research is needed to ensure services like Malabar can address issues like  smoking in pregnancy, address social and emotional issues facing Australian Aboriginal and Torres Strait Islander women and families. | | Further research is needed to ensure services like Malabar can resolve smoking in pregnancy, tackle social, and emotional challenges of Aboriginal and  Torres Strait Islander women and families. |
| Manniecon et al., (2003)  Australia | To analyze the perceptions, best practice models, expectations, and experiences of pregnancy, labor, and early post-partum period of young Indigenous mothers | 5 Aboriginal and Torres Strait Islander women | Qualitative study using semi-structured interviews | | This study highlighted the importance of family support throughout the entire birthing process and identified several barriers that exist for Aboriginal and Torres Strait Islander women accessing mainstream antenatal and postnatal services. | | The authors indicated that culturally sensitive antenatal classes, developing Indigenous specific health promotion strategies, provision of birth support, and cultural awareness training of healthcare workers are required. | | None suggested |
| Varcoe et al., (2013)  Canada | The purpose of this study was to understand rural Aboriginal women’s  experiences of maternity care, their desire for future care, and factors shaping those experiences and outcomes. | Over 100 Aboriginal women, men, youth, and elders  from four communities in | Critical Ethnographic Approach using observations, individual interviews, focus groups, and community | | The study revealed that Indigenous women's healthcare experiences were profoundly shaped by the intersection of rural geography, economic constraints, historical and ongoing colonization, racism, and a healthcare system that usually failed to respect the autonomy, cultural needs, and desire for family-centered care. | | The study emphasized that Aboriginal women and members of their communities  wanted choice and control. Also, rural Aboriginal communities and health organizations must collaborate to reform existing maternity services and support the return of birthing to rural and remote Aboriginal communities. | | None suggested |

**Table 4b**

*Summary of Quantitative Studies Included in the Scoping Review*

| **Author and**  **Country** | **Purpose/Aim** | **Population/**  **Sample** | **Methodology/**  **Methods** | **Findings/Healthcare Experiences** | **Implications** | **Areas for Future Research** |
| --- | --- | --- | --- | --- | --- | --- |
| Brown et al., (2015)  Australia | To compare the experiences of women who sought care at the AFBP^4^ and mainstream public antenatal care | 344 women who gave birth to an Aboriginal and or Torres Strait Islander baby | Cross-sectional Population-based Survey using a structured interview booklet | Women attending antenatal care services at an AFBP had a higher chance of reporting positive pregnancy experiences compared with those receiving mainstream care. This is because AFBP services are tailored, accessible, culturally responsive, offer social support, tailored, and rendered by Aboriginal health workers. | The study concluded that the AFBP effectively enhances the respondents' pregnancy experiences. Therefore, incorporating Aboriginal voices in antenatal care improves pregnancy experiences. | None suggested. |
| Brown et al., (2019)  Australia | To investigate the relationship between perceived discrimination and birth outcomes in perinatal care | 344 mother-infant dyads | Population-based study adopting the MIRE^1^ questionnaire | Overall, 1 in 2 Aboriginal women felt discriminated against compared to 1 in 4 non-Aboriginal women. The discrimination was in the form of feeling judged, talked down to, insulted, or ignored by healthcare staff. Mothers who felt discriminated against had an increased risk of poor outcomes. Also, respondents who reported social challenges or were very sick during pregnancy are more likely to report discrimination. | Clinicians need to understand the effects of intergenerational trauma on Aboriginal families. This would help to build trust between Aboriginal families and health staff. | Investigating cultural safety in Aboriginal perinatal care and developing strategies to reduce discrimination and improve the quality of Aboriginal prenatal care. |

^1^Measure of Indigenous Racism Experience (MIRE) (23)

*u*
